# A Fatal and Extremely Rare Obstetric Complication: Neglected Shoulder Presentation at Term Pregnancy

**DOI:** 10.1155/2015/819874

**Published:** 2015-08-05

**Authors:** Orkun Cetin, Halise Yolli, Numan Cim, Recep Yıldızhan, Hanım Guler Sahin

**Affiliations:** Department of Obstetrics and Gynecology, Faculty of Medicine, Yüzüncü Yıl University, 65080 Van, Turkey

## Abstract

Stillbirth is still an important problem for parents and healthcare providers worldwide. Nowadays, the neglected shoulder presentation is usually observed in developing countries and is associated with increased risk of fetomaternal morbidity and mortality. In recent years, there were limited reports about obstetric management of this serious complication in the literature. In this case report, we aimed at describing the neglected shoulder presentation at term pregnancy that caused fetal death and discussing management options for this rare obstetric complication during labor.

## 1. Introduction

Stillbirth is still an important problem for parents and healthcare providers worldwide. Perinatal mortality rates are over 60 per 1000 births in some low-income countries [1]. Intrapartum stillbirths account for nearly a quarter of all fetal deaths [2]. Although placental abruption, fetal distress, umbilical cord malformations, and malpresentations are the main causes of intrapartum stillbirths in term fetuses, several of these complications cannot be predicted during prenatal care [3, 4]. Maternal bleeding, abdominal pain, abnormal fetal heart rate pattern, and prolonged obstructed labor are some of the nonspecific signs of these complications in the antenatal period. An immediate caesarean section is the most common lifesaving treatment for the fetus and the mother in majority of cases [5, 6].

A transverse fetal position occurs approximately in one out of 300 deliveries [7, 8]. A neglected shoulder presentation is a rare form of persistent transverse fetal position. The major maternal and fetal risk factors for shoulder presentation during delivery include anatomic abnormalities of the pelvis, weakness of abdominal muscles, abnormalities of the uterus (bicornuate or septate), fibroids, pelvic masses, multiple gestations, polyhydramnios, placenta previa, prematurity, and intrauterine fetal demise (IUFD). This serious complication appears in a few cases due to lack of treatment for many hours during labor. In this condition, the fetal shoulder is impacted with the prolapsed arm, amniotic fluid is drained, uterus might be contracted, and the fetus is severely distressed or dead. Labor might progress spontaneously in smaller and macerated fetuses. In premature cases, labor might progress as the fetus becomes hyperflexed (defined as fetus condulicatus) and the fetal shoulder forces the pelvis, followed by the fetal head and trunk. However, this mechanism does not work in term pregnancies and larger fetuses.

Nowadays, the neglected shoulder presentation is usually observed in developing countries and is associated with increased risk of fetomaternal morbidity and mortality [9]. In recent years, there were limited reports about obstetric management of this serious complication in the literature. In this case report, we aimed at describing the neglected shoulder presentation at term pregnancy that caused fetal death and discussing management options for this rare obstetric complication during labor.

## 2. Case Report

**Figure 1:**
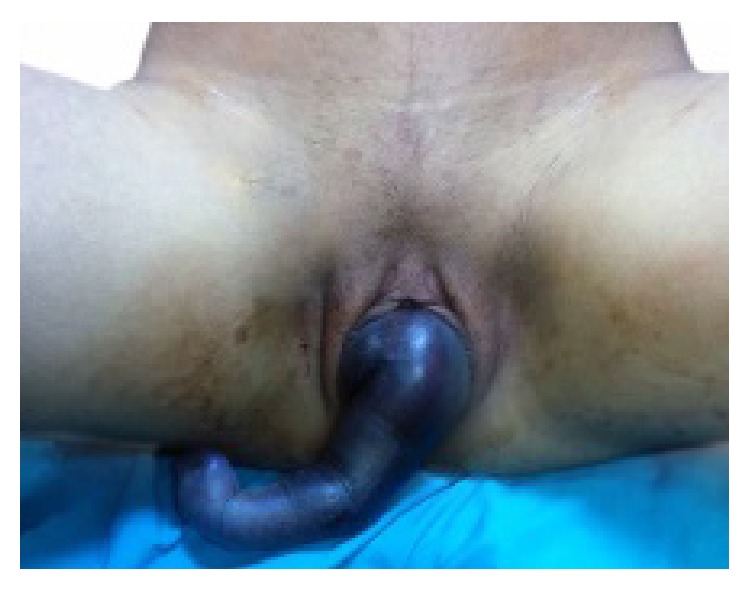
Neglected shoulder presentation at term pregnancy with intrauterine fetal demise. Cyanosed and prolapsed right fetal arm was seen outside of the vagina.

A 19-year-old, gravida 1, pregnant woman was referred to our tertiary hospital at 38 weeks of gestation with IUFD. The labor had begun at home eight hours ago with rupture of membranes. Duration of time from arm prolapse to hospital admission was approximately two hours. She was unsure for her date. On physical investigation, the prolapsed right arm (cyanosed) was seen outside of the vagina without cord pulsation, cervical dilatation was 7 cm, and finally the neglected shoulder presentation was observed (Figure 1). Upon ultrasound examination, no fetal heart activity was seen, the placenta had landed in the posterior part of the uterus, and the estimated fetal birth weight was 3000 g. The patient did not receive adequate prenatal care during pregnancy. Her medical history was unremarkable. At first, an attempt to take out the dead fetus by using internal podalic version and breech extraction was made, but this manipulation was inefficient. Decapitation was not chosen as a treatment option due to the clinician's insufficient experience and lack of proper instruments for this aggressive operation. Consequently, a caesarean section with low vertical incision was preferred as an alternative treatment method and a 2950 g, male, dead fetus were delivered. Prophylactic broad spectrum antibiotics were used to prevent uterine septicemia. No complications were recorded during the intraoperative and postoperative periods. The woman was discharged two days after the operation without any complication.

## 3. Discussion

In modern obstetrics, fetal size, fetal viability, rupture of the uterus, umbilical cord prolapse, previous caesarean section, and experience of the clinician are the most important factors that affect the management of fetal shoulder presentation during labor. Caesarean section, internal podalic version-breech extraction, and decapitation are the management modalities for neglected shoulder presentation. The viability of the fetus is the most important factor to be taken into consideration for a clinical decision [10].

Breech extraction following the internal podalic version under general anesthesia is the first option for small and nonviable fetuses. However, this procedure is associated with serious complications such as uterine rupture, bladder injury, and gross perineal lacerations. A high risk of uterine rupture is related to the thinning of the low uterine segment during prolonged labor. Therefore, the low uterine segment should be examined cautiously after delivery to rule out a possible rupture of the uterus [10]. In our case, the obstetrician tried to manipulate the fetus by using the internal podalic version as a first step but was not successful.

Decapitation could be preferred as a modality of treatment in dead fetuses for decreasing the surgical risks associated with caesarean section following prolonged labor. However, decapitation is an aggressive and radical procedure that could induce several injuries in genital and perineal organs such as uterus, bladder, cervix, and vagina [10]. Therefore, an experienced obstetrician should perform this procedure by using proper instruments. Decapitating hooks and Blond-Heidler saw are more useful instruments for this condition. Blond-Heidler is a braided wire saw which has a thimble introducer and two detachable handles. Decapitation has been well described by Lawson and Stewart in 1967 [11]. At first, the prolapsed fetal arm must be pulled down through the vagina for visualization of the fetal neck with the aid of an assistant. The Blond-Heidler saw is passed over the fetal neck and the thimble is connected. After chopping off the neck, the fetal trunk and head are delivered with traction [11]. Tocolytic therapy can be used for suppressing uterine contractility. The clinician should explore the genital tract cautiously to rule out any injuries after the procedure.

When the aforementioned manipulations for delivery fail, a caesarean section is the next step and last treatment option. If the baby is viable, this procedure must be preferred immediately. In our opinion, the main reason for choosing caesarean section in cases with nonviable fetuses is insufficient experience of the obstetrician regarding other procedures. Some obstetricians concluded that a caesarean section following prolonged labor is associated with several intraoperative and postoperative complications. The large size of the dead fetus, inefficient attempt of internal podalic version, and inadequate experience of the clinician on decapitation were the major factors that affected the obstetrician's decision in the present case. Septicemia is a frightening complication that could affect future fertility outcomes and cause maternal mortality [12]. Prophylactic broad spectrum antibiotics must be used routinely during and after surgery to decrease infective complications.

A neglected shoulder presentation is an extremely rare obstetric complication in developed countries; however, it is a reality in low-income parts of the world. Our tertiary care center is located in the rural and remote part of eastern Turkey. In this low-income region, many pregnant women deliver at home and go to the hospital only in case of emergency. This causes high rates of perinatal mortality. Therefore, clinicians who work in rural regions should be well equipped to handle such serious obstetric complications.

In cases of neglected shoulder presentation, a caesarean section should be preferred if the fetus is viable. It is also the safest approach if the obstetrician is not experienced regarding other procedures. In case of fetal death, decapitation by an experienced clinician is another choice. The clinicians should try to use the internal podalic version only in limited cases of neglected shoulder presentation.

## References

[1] World Health Organization (2007). *Neonatal and Perinatal Mortality: Country, Regional and Global Estimates 2004*.

[2] Lawn J. E., Blencowe H., Pattinson R. (2011). Stillbirths: where? When? Why? How to make the data count?. *The Lancet*.

[3] Thaddeus S., Maine D. (1994). Too far to walk: maternal mortality in context. *Social Science and Medicine*.

[4] Darmstadt G. L., Yakoob M., Haws R. A., Menezes E. V., Soomro T., Bhutta Z. A. (2009). Reducing stillbirths; interventions during labour. *BMC Pregnancy and Childbirth*.

[5] Goldenberg R. L., McClure E. M. (2009). Reducing intrapartum stillbirths and intrapartum-related neonatal deaths. *International Journal of Gynecology & Obstetrics*.

[6] Hofmeyr G. J., Hannah M. E. (2003). Planned caesarean section for term breech delivery. *Cochrane Database of Systematic Reviews*.

[7] Gemer O., Segal S. (1994). Incidence and contribution of predisposing factors to transverse lie presentation. *International Journal of Gynecology and Obstetrics*.

[8] Cruikshank D. P., White C. A. (1976). Obstetric malpresentations: twenty years' experience. *American Journal of Obstetrics & Gynecology*.

[9] Seffah J. D. (1999). Maternal and perinatal mortality and morbidity associated with transverse lie. *International Journal of Gynecology and Obstetrics*.

[10] Okonofua F. E. (2009). Management of neglected shoulder presentation. *BJOG: An International Journal of Obstetrics and Gynaecology*.

[11] Lawson J. B., Stewart D. B. (1967). Operations to relieve obstructed labour. *Obstetrics and Gynaecology in the Tropics and Developing Countries*.

[12] Aziken M. E., Omo-Aghoja L. O., Okonofua F. E. (2007). Perceptions and attitudes of pregnant women towards caesarean section in urban Nigeria. *Acta Obstetricia et Gynecologica Scandinavica*.

